# Aging in Place in Subsidized Housing: Roles and Perspectives of Staff Members

**DOI:** 10.1093/geroni/igaa057.797

**Published:** 2020-12-16

**Authors:** Sonia Pandit, Momana Jahan, David Reyes-Farias, Barbara Resnick, Carolina Reid, Rebecca Brown

**Affiliations:** 1 University of Pennsylvania, Philadelphia, Pennsylvania, United States; 2 University of Maryland School of Nursing, Baltimore, Maryland, United States; 3 University of California, Berkeley, Berkeley, California, United States

## Abstract

Nearly 3 million older Americans with low incomes live in subsidized housing. This population has disproportionate rates of functional impairment, cognitive impairment, and nursing home admission. Staff members who work in subsidized housing may have unique insight into how to improve aging in place for this vulnerable population, but little is known about their perspectives. We conducted 12 focus groups with 69 staff members from 7 subsidized housing sites. Staff included property managers, service coordinators, maintenance workers, administrative assistants, and security guards. Transcripts were analyzed using qualitative thematic analysis. The majority of participants noted that they “wear multiple hats” and their roles are not limited to professional responsibilities. Instead, their role is to maximize residents’ quality of life. As a result, staff members often adopt personal roles akin to serving as residents’ “surrogate family” members. This relationship gives staff a unique window into residents’ lives which enables them to detect early warning signs among residents. For example, staff often observed changes in residents’ physical appearance and hygiene, mood, behavior, and function which could indicate physical illness, mental illness, or cognitive impairment. Staff reported having some resources to address these warning signs, such as involving families and connecting residents to community resources. However, they also highlighted unmet needs to address these issues, such as on-site clinical staff and affordable mental health services. Our findings suggest that building staff are a valuable and underutilized resource for identifying at-risk residents in subsidized housing and helping to deliver interventions to improve aging in place.

